# Vertical Inheritance Facilitates Interspecies Diversification in Biosynthetic Gene Clusters and Specialized Metabolites

**DOI:** 10.1128/mBio.02700-21

**Published:** 2021-11-23

**Authors:** Alexander B. Chase, Douglas Sweeney, Mitchell N. Muskat, Dulce G. Guillén-Matus, Paul R. Jensen

**Affiliations:** a Center for Marine Biotechnology and Biomedicine, Scripps Institution of Oceanography, University of California San Diego, La Jolla, California, USA; b Center for Microbiome Innovation, University of California San Diego, La Jolla, California, USA; University of Maryland School of Medicine

**Keywords:** *Salinispora*, salinosporamide, homologous recombination, microbial ecology, evolution, evolutionary biology

## Abstract

While specialized metabolites are thought to mediate ecological interactions, the evolutionary processes driving chemical diversification, particularly among closely related lineages, remain poorly understood. Here, we examine the evolutionary dynamics governing the distribution of natural product biosynthetic gene clusters (BGCs) among 118 strains representing all nine currently named species of the marine actinobacterial genus *Salinispora*. While much attention has been given to the role of horizontal gene transfer (HGT) in structuring BGC distributions, we find that vertical descent facilitates interspecies BGC diversification over evolutionary timescales. Moreover, we identified a distinct phylogenetic signal among *Salinispora* species at both the BGC and metabolite level, indicating that specialized metabolism represents a conserved phylogenetic trait. Using a combination of genomic analyses and liquid chromatography–high-resolution tandem mass spectrometry (LC-MS/MS) targeting nine experimentally characterized BGCs and their small molecule products, we identified gene gain/loss events, constrained interspecies recombination, and other evolutionary processes associated with vertical inheritance as major contributors to BGC diversification. These evolutionary dynamics had direct consequences for the compounds produced, as exemplified by species-level differences in salinosporamide production. Together, our results support the concept that specialized metabolites, and their cognate BGCs, can represent phylogenetically conserved functional traits with chemical diversification proceeding in species-specific patterns over evolutionary time frames.

## INTRODUCTION

While linkages have been established between the abiotic factors structuring bacterial diversity (1–3), the key functional traits driving biotic interactions among microbes remain poorly understood. These traits likely include the production of antibiotics, siderophores, and other specialized metabolites that could play major yet poorly defined roles in mediating microbial interactions. The ecological functions of specialized metabolites include, among others, allelopathy, nutrient uptake, and defense against predation (4), all of which can have a major effect on microbial community composition (5). To date, however, studies of bacterial specialized metabolite production have largely focused on the discovery of compounds with pharmaceutical potential as opposed to understanding their ecological functions or the evolutionary processes contributing to chemical diversification.

Although it remains difficult to identify the ecological roles of specialized metabolites (6), the evolutionary processes governing their diversity and distribution can be inferred from the biosynthetic gene clusters (BGCs) that encode their production. Several comparative genomic studies have identified horizontal gene transfer (HGT) as an integral driver of BGC evolution (7–10). Under this evolutionary model, the exchange of BGCs among disparate strains would result in poor correlations between BGC distributions and species phylogenies. Vertical inheritance is also expected to influence BGC evolutionary dynamics, which is evident from BGC conservation among closely related strains (11–14). Likely, a combination of HGT and vertical inheritance contributes to the collection of BGCs observed among most strains (15). This may include instances where both processes act on the same BGC, as in the case of horizontally acquired BGCs that are subsequently maintained in extant lineages through vertical descent. While much attention has focused on the propensity for HGT, vertical inheritance provides an alternative framework to evaluate the evolutionary processes contributing to BGC diversification and their effects on specialized metabolite production.

Here, we coupled comparative genomics with targeted metabolomics to assess the evolutionary processes structuring BGC diversity and their effects on specialized metabolite production in the marine actinobacterial genus *Salinispora* (family, *Micromonosporaceae*). While a member of the rare biosphere (16), *Salinispora* strains can be readily cultured from tropical and subtropical marine sediments (17) and other marine sources (18). Along with other marine sediment-inhabiting *Actinobacteria* (19–21), the genus is well-known for the production of specialized metabolites. While *Salinispora* has proven a robust model for natural product discovery (22), much remains to be resolved concerning BGC evolution and its effects on specialized metabolite diversification in this taxon. For instance, prior analyses revealed extensive HGT of *Salinispora* BGCs (23), suggesting a “plug-and-play” model for the acquisition of new BGCs (24). However, the production of specific specialized metabolites were constrained to single species (25), indicating that vertical inheritance also plays an important role in BGC evolution.

To better understand the relative roles of HGT and vertical inheritance in the genus *Salinispora*, we revisited the distribution of BGCs in 118 genomes across the nine described species (26). Given that all of these *Salinispora* strains share >99% 16S rRNA sequence identity, and thus are estimated to have diverged <50 million years ago (27), this genomic data set allows for the identification of evolutionary processes that could be masked by similar analyses of more divergent taxa (28). We hypothesized that the collection of BGCs, even if they were horizontally acquired at some point in time (23), would exhibit a strong phylogenetic signal indicating that vertical inheritance is a major driver of BGC evolution. We further expected that evolutionary processes would promote interspecies BGC diversification and that these would be reflected in the compounds produced. To associate BGC diversification with compound production, we focused on nine experimentally characterized BGCs and applied targeted tandem mass spectrometry to detect their associated small-molecule products. Our results support the concept that specialized metabolites vary in their degree of phylogenetic conservation with some likely to represent key functional traits associated with species diversification.

## RESULTS

### *Salinispora* spp. delineated by biosynthetic potential.

**FIG 1:**
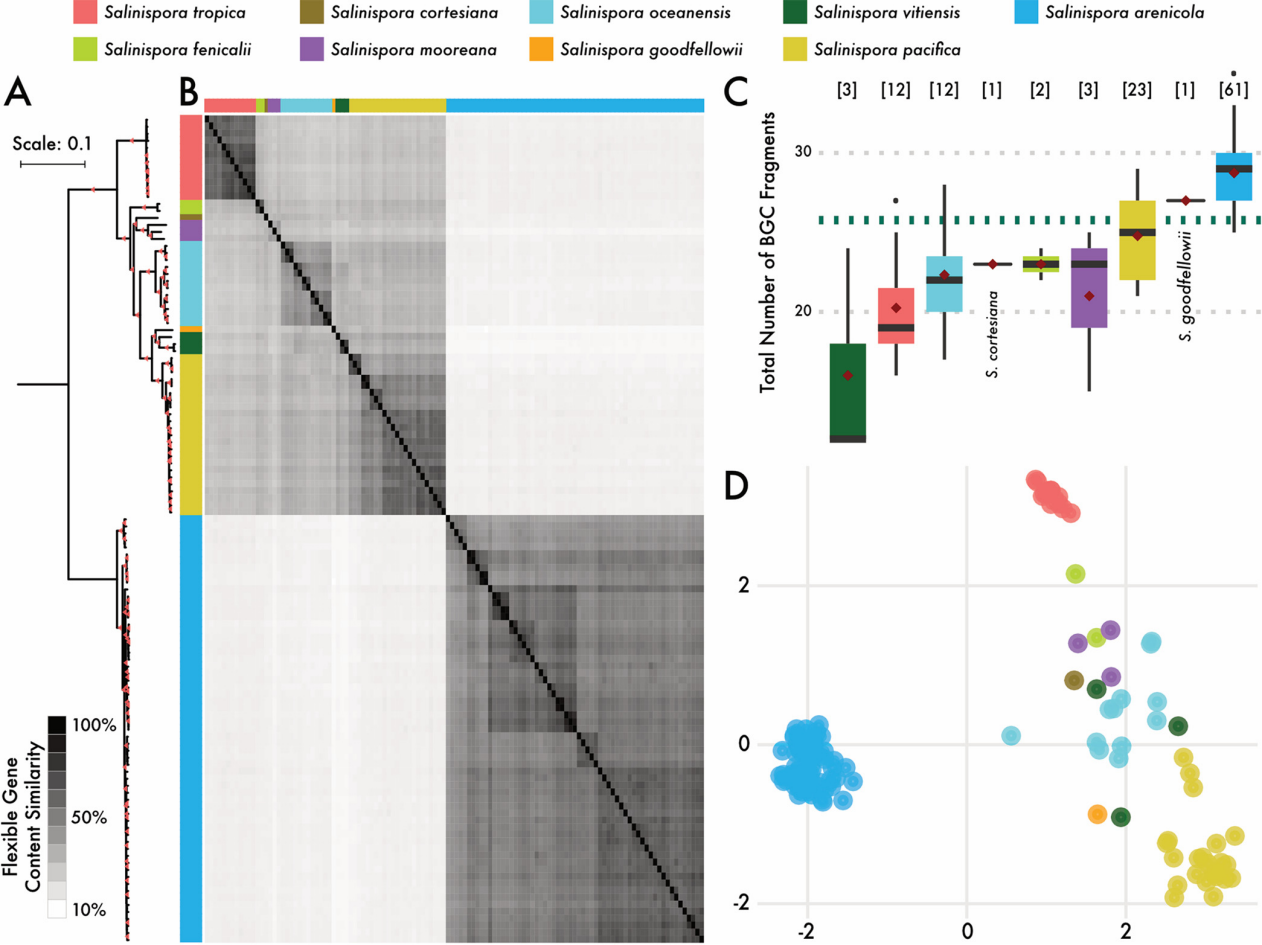
Genetic relatedness of *Salinispora* strains (*n* = 118). (A) Core genome phylogeny. Colors denote species. Bar, 0.1 nucleotide substitutions per position. Red triangles indicate nodes with bootstrap support >90%. (B) Heatmap depicting pairwise similarities in flexible genome content among strains (generated from a Jaccard distance matrix). (C) Total number of biosynthetic gene cluster (BGCs; both whole and fragments) identified across *Salinispora* species. Black bars represent medians, red diamonds represent means, and dashed green line denotes average across the genus. “N” indicates number of genomes per species. (D) Nonmetric multidimensional scaling (NMDS) plot depicting biosynthetic gene cluster family (GCF) composition for each *Salinispora* strain. Circles represent strains colored by species.

To identify the traits differentiating the nine *Salinispora* species, we investigated differences in gene content (i.e., the flexible genome) across 118 genomes (Table S1 in the supplemental material). Similarity in flexible gene content among strains was highly congruent with the core genome phylogeny (Fig. 1A and B); strains within the same species shared more flexible genes than expected by chance, with 53.1% of the total variation explained by species designation (Table S2) (permutational multivariate analysis of variance [PERMANOVA], *P < *0.01). Geographic location only accounted for 8.4% of the variation (Table S2) (*P < *0.01). Within the flexible genome, we also identified genes shared by all strains within a species, but not observed in any other *Salinispora* species, and defined these as species-specific orthologs. These genes, which are predicted to encode functional traits that delineate *Salinispora* species, were largely annotated as hypothetical proteins (Fig. S1A at https://osf.io/entqc/); however, 18.1 ± 15.3% of annotations were associated with specialized metabolism (Fig. S1B). Moreover, when we searched the genomic regions flanking these species-specific orthologs, regardless of annotation, 28.9 ± 26.2% were located within the boundaries of predicted biosynthetic gene clusters (BGCs; Fig. S1C at the URL mentioned above).

10.1128/mBio.02700-21.1 TABLE S1 Genomic and geographic characteristics of *Salinispora* strains. Download Table S1, XLSX file, 0.02 MB. Copyright © 2021 Chase et al. 2021 Chase et al. https://creativecommons.org/licenses/by/4.0/ This content is distributed under the terms of the Creative Commons Attribution 4.0 International license.

10.1128/mBio.02700-21.2 TABLE S2 PERMANOVA results for the effects of species, geography, and species geography interaction for flexible genome (A), gene cluster family (GCF) (B), metabolome (C), and analog composition (D). Download Table S2, XLSX file, 0.01 MB. Copyright © 2021 Chase et al. 2021 Chase et al. https://creativecommons.org/licenses/by/4.0/ This content is distributed under the terms of the Creative Commons Attribution 4.0 International license.

Given the high percentage of species-specific flexible genes associated with specialized metabolism, we expected that BGC distributions would similarly correspond with *Salinispora* species diversity. To address this, we identified a total of 3,041 complete or fragmented (on contig edges) BGCs across all 118 *Salinispora* genomes (mean, 25.8 per genome) accounting for 18 ± 2.3% of an average 5.6-Mbp *Salinispora* genome. Between *Salinispora* species, there was significant variation in the total number of BGCs (Fig. 1C) (analysis of variance [ANOVA], *P < *0.001) and the genomic percentage dedicated to specialized metabolite production (ANOVA, *P < *0.001). In cases where the number of genome sequences is low (e.g., Salinispora vitiensis), BGC abundances may not be fully representative of that species.

To compare BGC composition across species, the 3,041 predicted BGCs were grouped into 305 gene cluster families (GCFs) (Fig. S2A at https://osf.io/entqc/). Similar to prior reports (24), 35% of these GCFs were only observed in one strain (Fig. S2B at the URL mentioned above); however, these singletons account for only 108 of the total 3,041 BGCs detected among all strains (3.6%). Importantly, this equates to an average of 0.9 singleton BGCs per *Salinispora* genome (Fig. S2B at the URL mentioned above, inset), indicating that only a small proportion of the BGCs in each *Salinispora* genome (0.9 out of 25.8 BGCs per genome) represent relatively recent acquisition events. Conversely, the majority of BGCs were shared among *Salinispora* strains (Fig. S2A and C at the URL mentioned above) such that 43.6% of the variation in GCF composition was explained by species designation (Fig. 1D; Table S2) (PERMANOVA, *P < *0.01), with geography explaining an additional 11.1% (*P < *0.01), mirroring the results from the flexible genome analysis. Correlations between GCF distributions and species delineations were further supported by BGC average nucleotide identity (ANI) values, which resembled whole-genome ANI values except for a small percentage (1.4%) that showed evidence of relatively recent interspecific horizontal transfer (Fig. S3 at the URL mentioned above). Taken together, these results indicate that HGT provides a mechanism to expand GCF diversity within the genus, while species-level GCF composition is largely constrained by shared phylogenetic history.

### Drivers of GCF evolution.

**FIG 2:**
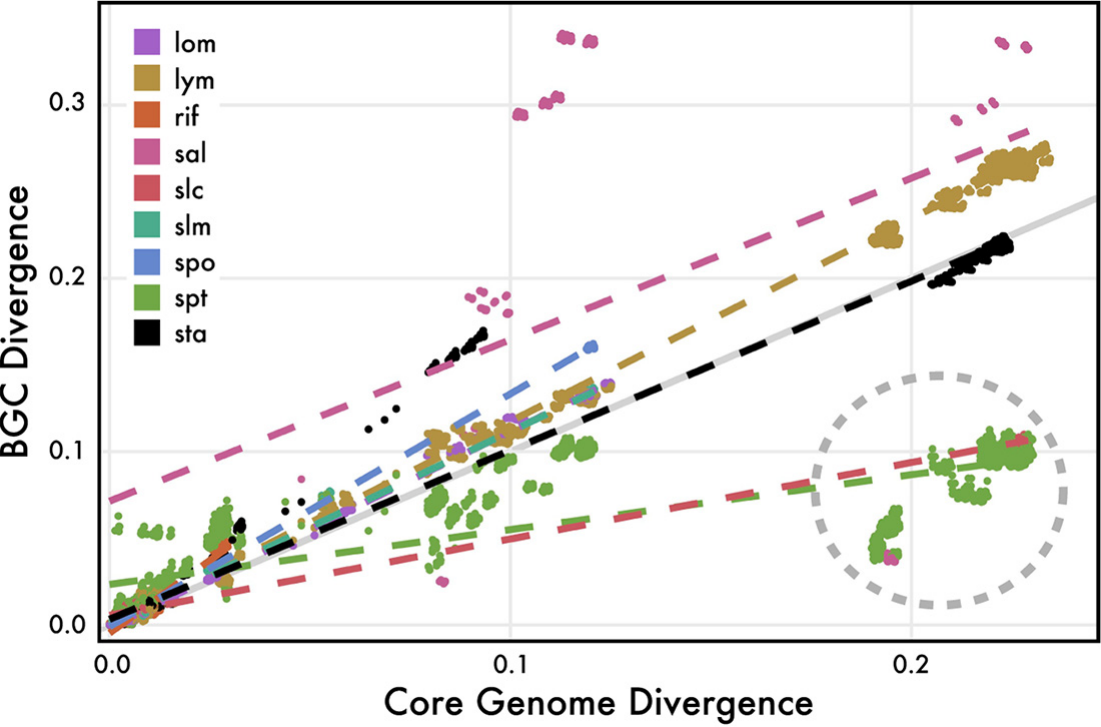
Gene cluster family (GCF) genetic similarity since divergence from a common ancestor (inferred from core genome divergence). Each point represents a pairwise comparison calculated from whole-BGC alignments as a function of core genome divergence for the same strain pairs. Linear regression lines are denoted for each GCF, with the regression line for expected neutral divergence (i.e., diverging at the same rate as the core genome) in gray. Predicted interspecies transfer events are circled in gray for *sal*, *slc*, and *spt* (see Fig. S4A at https://osf.io/entqc/).

We next sought to identify the specific evolutionary processes that contributed to the GCF distributions observed within the genus. To so do, we concentrated on nine experimentally characterized BGCs (Table S3) and their associated GCFs (29–37). These GCFs range in conservation from species specific to nearly ubiquitous in the genus. The more conserved GCFs (i.e., *lym*, *sta*, and *spt*) predate *Salinispora* speciation events, while others (i.e., *slc*) were acquired more recently (Fig. S4A at https://osf.io/entqc/). Despite these differences, the phylogenies of all nine GCFs (Fig. S4B at the URL mentioned above) revealed that genetic differentiation remained a function of divergence time and is comparable to core genome measurements (Fig. 2) (multiple *r*^2^ = 0.84, *P < *0.001). Thus, even in cases where the BGC may have originally been acquired through HGT, the genetic diversity within all nine GCFs was structured and subsequently maintained by processes of vertical descent over evolutionary timescales.

10.1128/mBio.02700-21.3 TABLE S3 Additional information on nine BGCs and essential biosynthetic genes. Download Table S3, XLSX file, 0.01 MB. Copyright © 2021 Chase et al. 2021 Chase et al. https://creativecommons.org/licenses/by/4.0/ This content is distributed under the terms of the Creative Commons Attribution 4.0 International license.

An event inference parsimony model indicated that a variety of evolutionary processes, including BGC duplication, transfer, and loss, contributed to the distributions of the nine GCFs (Table 1). All nine GCFs were predicted to follow a strict model of vertical inheritance with frequent intraspecific recombination (18.7 ± 14.7 recombination events per GCF) contributing to species-level coherence. Conversely, GCF transfer between species was relatively rare (0.9 ± 1.1 HGT events per GCF). Even in cases where interspecies transfers were detected (e.g., *sal* and *slc*), genetic differentiation within each GCF remained a function of divergence time (Fig. 2), supporting a single ancestral transfer event followed by vertical inheritance. The model further revealed that BGC loss events (Table 1) explained the patchy distributions of the conserved GCFs. For instance, the *lom* GCF followed a strict model of vertical inheritance with predicted loss events in S. mooreana and S. oceanensis (Fig. S4B at https://osf.io/entqc/). Overall, frequent intraspecies recombination and loss events, along with rare interspecies HGT events, allowed for *Salinispora* GCFs to diverge in a species-specific pattern.

**TABLE 1:** Recombination, transfer, duplication, and loss events of nine GCFs relative to the core genome^*a*^

BGC	No. of species	No. of strains	Alignment length (bp)	Recombination metrics				DTL model-predicted events			
				*R*/θ	δ	ν	*r*/*m*	*T* (inter)	*T* (intra)	*D*	*L*
*lom*	6	42	84,964	0.016	6,696.0	0.026	2.77	0	10	0	14
*lym*	9	109	32,597	0.019	4,101.2	0.021	1.63	0	33	1	33
*rif*	1	59	91,550	0.655	6,368.3	0.003	13.90	0	23	0	15
*sal*	6	30	15,911	0.024	3,453.9	0.032	2.70	2	5	0	16
*slc*	2	25	31,937	0.020	1,316.7	0.058	1.57	1	11	1	5
*slm*	3	25	28,111	0.189	428.0	0.041	3.28	1	10	0	14
*spo*	2	13	167,237	0.018	1,032.6	0.093	1.71	1	5	0	3
*spt*	7	112	13,368	0.904	1,115.5	0.010	10.16	3	49	0	22
*sta*	4	77	19,319	0.069	1,352.4	0.032	2.96	0	22	1	23
Core genome	9	28	4,668,606	0.061	1,111.6	0.026	1.75				

a *R*/θ, relative rate of recombination to mutation; δ, mean import length of recombining DNA; ν, mean divergence of imported recombining DNA; *r/m*, relative impact of recombination to mutation accumulation on per-site substitution rate (equal to R/θ × δ × ν); DTL, duplication, transfer, and loss.

Given evidence of vertical maintenance in the nine GCFs over evolutionary time, we next investigated the evolutionary processes that can alter the genetic diversity within these BGCs. Specifically, we focused on the ratio at which nucleotides are replaced by either recombination or point mutations (*r/m*). At the genome level, the high levels of recombination (Table 1) (*r/m *= 1.8) observed among closely related strains (ν = 0.03 or 3% genetic divergence among strains) maintains genetic cohesion within species. This was further supported by a recombination network that revealed no recent gene flow between *Salinispora* species (Fig. S5 at https://osf.io/entqc/). Interestingly, the detection of isolated recombining populations within species, some of which are location specific, may provide evidence of nascent speciation events. Similarly, the high levels of recombination detected across all nine GCFs (all *r/m *> 1.5) were restricted to closely related strains (ν_MEAN_ = 3.5%) (Table 1). For example, recombination in the *rif* BGC (*r/m *= 13.9) was restricted to strains that have diverged by <0.3%. These events were also restricted in the size of the recombining fragments (δ), as small sections of the BGC were exchanged rather than the entire cluster (Table 1). Finally, recombination had various effects on the genetic diversity of the nine GCFs. For instance, the two most widely distributed GCFs, *lym* and *spt*, exhibited drastically different *r/m* values (1.6 and 10.2, respectively). Correspondingly, reduced recombination allowed the *lym* GCF to evolve in accordance with the core genome, while frequent recombination of the *spt* GCF limited the number of polymorphisms (Fig. 2). Over evolutionary timescales, recombination of small sections of BGCs can homogenize genetic diversity among closely related strains while accelerating BGC diversification between species.

Increased recombination can also facilitate gene-specific selective sweeps (38), which can be evident from reduced nucleotide diversity within biosynthetic genes compared to that of whole-genome measurements. A comparison of the conserved biosynthetic genes found in the nine GCFs revealed reduced nucleotide diversity in the *sal* and *spo* GCFs in S. pacifica and the *slc* in S. arenicola (Fig. S6A at https://osf.io/entqc/). However, the GCFs in these three instances were only observed in a small number of closely related strains (i.e., three S. pacifica and five S. arenicola strains sharing >99.6% and >99.4% genome-wide ANI, respectively), which likely accounts for the reduced diversity. In contrast, most conserved biosynthetic genes showed no evidence of recent selective sweeps, with the relatively high nucleotide diversity indicating that recombination was insufficient to prevent GCF diversification, a pattern that is further evidenced by the absence of interspecies gene flow in the recombination network (Fig. S5 at the URL mentioned above). Notably, this diversification was indicative of neutral or purifying selection, with 97.8% of the 134 conserved biosynthetic genes analyzed having a ratio of nonsynonymous to synonymous evolutionary changes (*dN*/*dS*) of <1 (Fig. S6B at the URL mentioned above).

### Specialized metabolites as functional traits.

**FIG 3:**
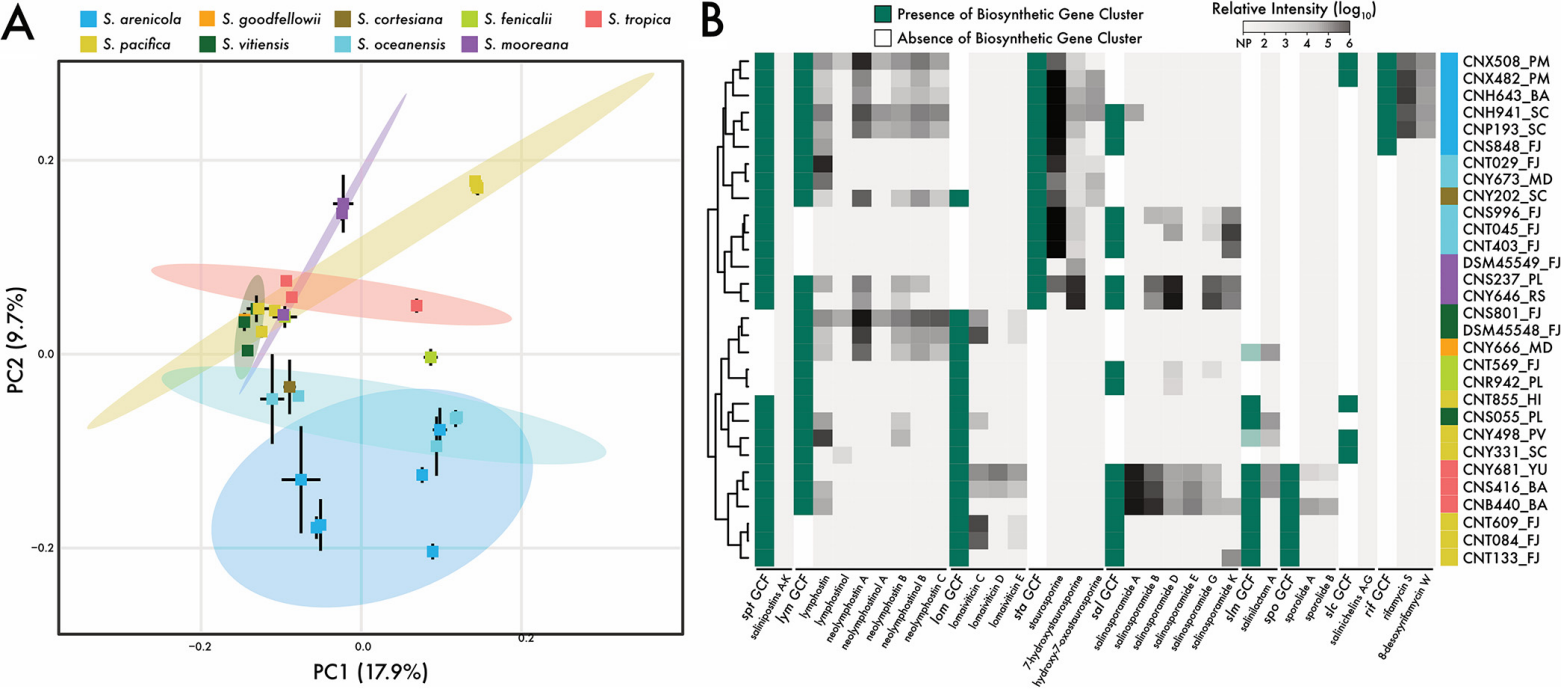
*Salinispora* metabolomics and GCF distributions across nine species (*n* = 30 strains). (A) Principal-components analysis (PCA) showing differences in metabolomes. Each point represents a strain colored by species. Standard deviations are shown as black lines for each strain, and 95% confidence intervals are denoted as colored ellipses around each species with ≥3 strains. (B) Targeted analysis for the products of nine GCFs. Hierarchical clustering generated from a Euclidean distance matrix. Green/white columns show the BGC presence/absence within a GCF. Light green indicates the detection of a BGC fragment. Gray/black columns show the relative production of each identified analog, NP, no production. *x* axis lists GCF name followed by product names. *y* axis lists strains colored by species with abbreviations for geographic origin (see Table S1 in the supplemental material for more information on geographic locations).

To address the relationships between BGC evolution and specialized metabolite production, we applied LC-MS/MS to culture extracts from 30 representative strains across all nine *Salinispora* species. Differences in metabolic features were greater between species than within the same species (Fig. 3A; Table S2) (PERMANOVA, *P < *0.01), with 44.9% of the variation explained by species designation. This provides evidence at the compound level that vertical descent plays a major role in structuring *Salinispora* specialized metabolism. Next, we applied targeted LC-MS/MS analyses to test for linkages between genetic polymorphisms observed within the nine GCFs and the compounds they encode (Fig. S7 at https://osf.io/entqc/). In total, products from seven of the nine GCFs were detected (Fig. 3B; Table S4), with culture conditions and/or extraction methods likely accounting for the two that were not (*slc* and *spt*). Strains from different species produced significantly different compound analogs (Fig. 3B; Table S2) (PERMANOVA, *R*^2^ = 0.68, *P* < 0.01), indicating that differences between species were greater than those within. Interestingly, a high degree of similarity in the rifamycins detected across S. arenicola strains was observed (Fig. 3B) despite the high level of nucleotide diversity that has accumulated in the *rif* GCF (Fig. S6A at the URL mentioned above), suggesting these genetic changes are largely neutral in terms of compound production.

10.1128/mBio.02700-21.4 TABLE S4 Molecular targets detected at <10 ppm error from theoretical mass. Feature IDs correspond to extracted features from MS1 analysis. Δppm averaged across strains. Positive dereplication source indicated in the dereplication column. “GNPS library” indicates a minimum of 4 matched peaks to an entry in the GNPS library; “GNPS network” indicates a node connected GNPS library hit or internal standard in the molecular network. ND, not detected. Download Table S4, XLSX file, 0.2 MB. Copyright © 2021 Chase et al. 2021 Chase et al. https://creativecommons.org/licenses/by/4.0/ This content is distributed under the terms of the Creative Commons Attribution 4.0 International license.

Interspecies genetic differentiation within shared GCFs could be linked to differences in compound production. For example, three of the four *Salinispora* spp. that maintain the *sta* GCF all produce staurosporine, while the fourth species, S. mooreana, preferentially produced 7-hydroxystaurosporine (Fig. 3B). Comparative genomics revealed that the version of the *sta* BGC in S. mooreana lacks the NAD-dependent dehydratase gene (Fig. S8A at https://osf.io/entqc/) that likely accounts for the hydroxy analog produced by this species (Fig. S7 at the URL mentioned above). More pronounced interspecies polymorphisms were observed in the *spo* GCF between S. tropica and the subset of S. pacifica strains (3 of 23) that possess the BGC. While all strains maintain the type I polyketide synthase (PKS) genes responsible for the polyketide core (37), the three S. pacifica strains lack the 45-kbp nonribosomal peptide synthetase (NRPS) genes, which encode the biosynthesis of the cyclohexenone epoxide subunit (Fig. S8B at the URL mentioned above). Correspondingly, these S. pacifica strains did not produce sporolides (Fig. 3B) or any identifiable derivatives. Interestingly, the 20 S. pacifica strains that lack the *spo* GCF encode a similar enediyne GCF linked to cyanosporaside production (23), suggesting the products may perform similar ecological functions. Overall, the effects of vertical inheritance on *Salinispora* GCF diversification had direct implications for species-level specialized metabolite production.

### Salinosporamides: a case study for BGC evolution.

**FIG 4:**
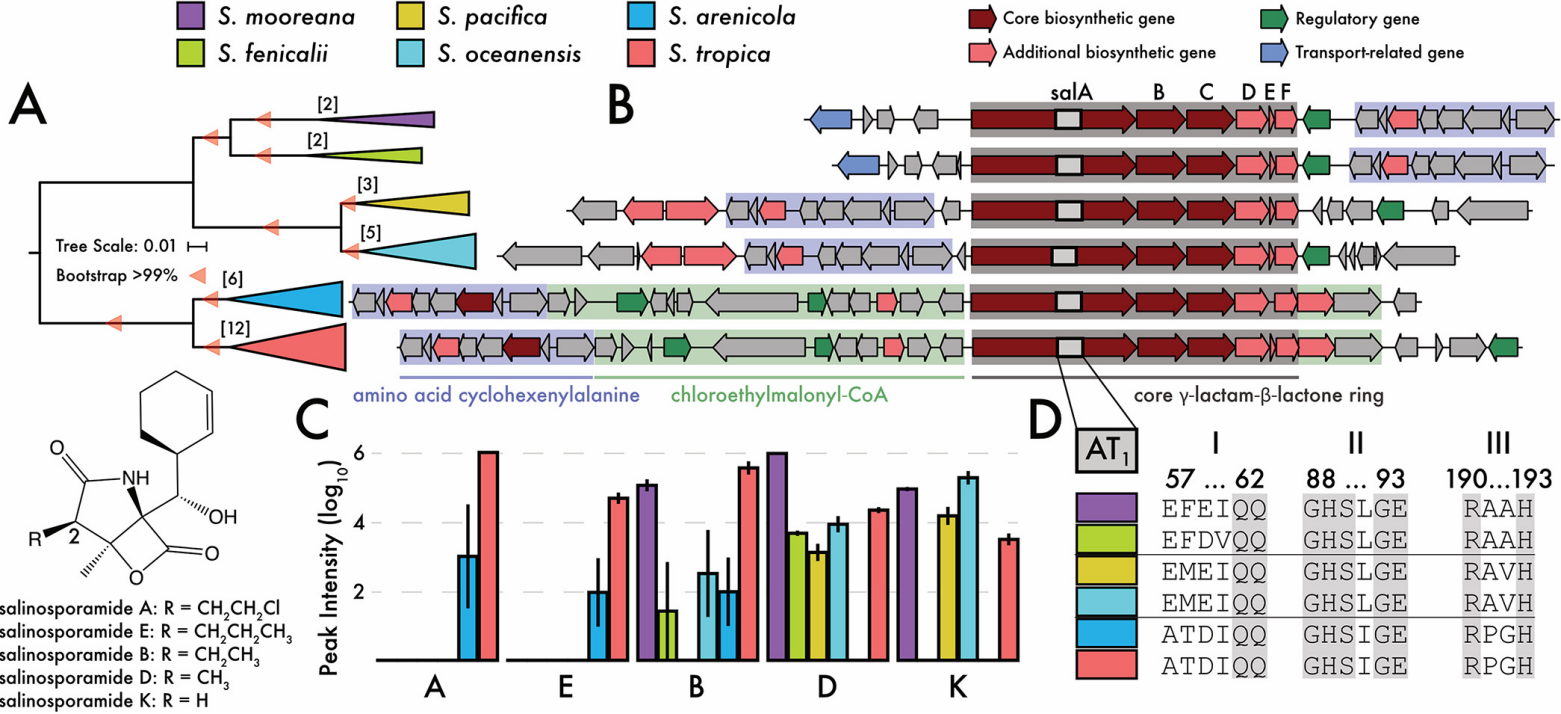
Variations in the salinosporamide GCF (*sal*) and its products. (A) Phylogeny of the *sal* BGC across species. Colors denote species. Bar, 0.01 nucleotide substitutions per position. Bootstrap values indicate >99% support. Brackets indicate number of genomes in each species encoding a version of the BGC. (B) Representative BGC for each species. Genes are colored by predicted biosynthetic function with gene blocks colored by their role in compound production. (C) Salinosporamide production across species. Analogs are listed in descending order based on R group size (inset). (D) Sequence alignments of the *salA* AT_1_ domain showing three signature motifs associated with substrate specificity. Conserved amino acid regions in gray.

To further illustrate the evolutionary processes contributing to chemical diversification, we examined the *sal* GCF, which encodes the biosynthesis of the anticancer agent salinosporamide A and analogs (29, 39). While salinosporamides A and K were originally reported from S. tropica (40) and S. pacifica (39, 41), respectively, we now show that versions of the *sal* BGC occur in six of the nine *Salinispora* species (Fig. 4A). Phylogenetic analysis provides evidence that this GCF was transferred between S. arenicola and S. tropica but has otherwise descended vertically within the genus (Fig. S4A at https://osf.io/entqc/). Notably, the *sal* GCF is rapidly diverging compared to the core genome (Fig. 2), while, at the same time, gene conservation and synteny are highly conserved at the species level (Fig. S9 at the URL mentioned above). For this reason, the *sal* GCF provides a useful model to address the relationships between species diversification, BGC composition, and compound production.

All strains encoding the *sal* GCF maintain the biosynthetic genes responsible for the core γ‐lactam‐β‐lactone ring (*salABCDEF*) and the cyclohexenylalanine amino acid residue, although the position of the genes for the latter varied (Fig. 4B). While 15 of 16 strains produced detectable amounts of salinosporamides, the analogs and their yields varied across species (Fig. 4C) (ANOVA, *P < *0.01). S. tropica and S. arenicola were the only species in which salinosporamide A was detected, and they are the only species to possess the 15-kbp region of the BGC responsible for assembly of the chloroethyl-malonyl‐CoA extender unit (Fig. 4B) (29). This is the first report of salinosporamide A production in S. arenicola, although the presence of the BGC in this species was previously documented (24, 42). While the versions of the *sal* BGC in S. tropica and S. arenicola are nearly identical (e.g., *salA* and *salB* genes share 95.1% and 97.5% amino acid sequence identity between species, respectively), salinosporamide production in S. arenicola was dramatically reduced relative to S. tropica (Fig. 4C, note log scale). This could be due to a splicing event in the overlapping coding regions in *salD/E* (Fig. 4B), which may render *salE*, which encodes a chaperone MbtH-like protein in NRPS biosynthesis (43), inactive.

Molecular networking provided additional insights into species-specific patterns of salinosporamide production (Fig. S10 at https://osf.io/entqc/). Here, we detected potentially new salinosporamide derivatives, including one (*m/z* 307.154) produced exclusively by S. mooreana. Inversions of the cyclohexenylalanine amino acid-encoding region of the *sal* BGC in S. mooreana and S. fenicalii (Fig. 4B) could facilitate structural modifications by downstream auxiliary genes (Fig. S9 at the URL mentioned above). The network also revealed evidence that salinosporamide analog production varied by species. As mentioned, S. tropica and S. arenicola were the only species to produce salinosporamides A and E, which incorporate chloroethylmalonyl-CoA or propyl malonyl‐CoA extender units, respectively (29), resulting in the largest R groups at the C-2 position of the γ-lactam side chain in this compound class (Fig. 4C). S. fenicalii and S. mooreana preferentially produced salinosporamides B and D, which possess medium-sized R groups, while S. oceanensis and S. pacifica preferentially produce salinosporamide K, which has the smallest R group.

Based on these observations, we suspected substrate discrimination by the *salA* PKS acyltransferase domain (AT_1_). The three signature motifs related to extender unit specificity (39) were highly conserved at the species level and strongly associated with the length of the C-2 R group in the salinosporamide product (Fig. 4D; Fig. S11 at https://osf.io/entqc/). Structural protein modeling of the AT_1_ domain identified motif I (amino acid residues 57 to 59) as a novel extension of the active site that could allow for extender unit discrimination (Fig. S12A at the URL mentioned above). In this case, the smaller, polar residues (Ala57 and Thr58) in S. tropica and S. arenicola (Fig. S12B at the URL mentioned above) could accommodate larger substrates yielding salinosporamides A and E, while the larger residues observed at these positions in other species may not. Thus, salinosporamide production and its associated bioactivity (i.e., salinosporamide A is 100× more cytotoxic than salinosporamide K [39]) is likely affected not only by the ability to synthesize the various extender units (29) but also selection of the appropriate substrate by the AT_1_ domain.

## DISCUSSION

It has become increasingly clear that the fine-scale diversity observed in microbial communities reflects a large number of co-occurring and ecologically distinct lineages (44–47). At the same time, the functional traits that differentiate closely related lineages, thus allowing for niche partitioning, remain less clear (48, 49). By analyzing 118 closely related strains (i.e., >99% similarity in the 16S rRNA gene) representing 9 species within the marine actinobacterial genus *Salinispora*, we linked interspecies genetic diversity to trait variation in the form of specialized metabolism. Specifically, the flexible genome, which encodes genes related to fitness differences (50), revealed species-specific signatures in BGC composition that translated into similar patterns of specialized metabolite production. Notably, vertical inheritance over long evolutionary timescales facilitated BGC diversification among *Salinispora* species that could be linked to the generation of new chemical diversity.

In plants and animals, the production of specialized metabolites can be phylogenetically conserved (11, 51) insomuch as they can be used as chemotaxonomic markers (52). In bacteria, compound production has been linked to taxonomy (53) and phylogenetic history (54), and there is mounting evidence of the importance of vertical inheritance in BGC evolution (15, 55). Yet the relative roles of vertical descent and HGT in mediating BGC evolution remain poorly understood for most bacteria. In this study, we identified a distinct phylogenetic signal among *Salinispora* species at both the BGC (Fig. 1D) and metabolite (Fig. 3) levels. While few BGCs were conserved across all species (e.g., *lym*), evidence that compound production was conserved at the species level (Fig. 3A) supports the concept that *Salinispora* specialized metabolites are functional traits associated with ecological differentiation (56). Indeed, our prior work with two *Salinispora* species revealed distinct ecological strategies related to resource acquisition and the production of antibiotics (4). Evidence of species-level specialized metabolite conservation suggests that the production of these compounds, many with documented biological activities, can provide a selective advantage by mediating biotic interactions in sediment communities (57).

Our results support prior studies that HGT plays a major role in expanding the diversity of BGCs observed within the genus *Salinispora* (23, 24). Yet most BGCs within each of the 118 *Salinispora* genomes were vertically retained over evolutionary timescales. Within a more diverse group of *Streptomyces* strains, it was recently estimated that a single gene is acquired through HGT every 100,000 years (28), whereas BGCs are retained in specific lineages over millions of years through vertical evolution (15). Determinations of the relative impact of HGT in structuring BGC diversity are highly dependent on the number of strains examined and their genetic relatedness. For instance, it could be presumed that a BGC observed in only one strain is a relatively recent acquisition event, while the inclusion of more closely related strains may indicate that this event occurred long ago in the evolutionary history of the lineage. Furthermore, BGC distributions are often described by their presence or absence without considering the degree of genetic divergence, which is a function of divergence time. Additional work considering BGC diversity among large strain collections will advance our understanding of the rates and frequencies of HGT events and their role in BGC evolution (55).

A closer examination of nine *Salinispora* gene cluster families (GCFs), which ranged in conservation from species to genus specific, revealed a variety of evolutionary processes impacting BGC genetic diversification and, ultimately, specialized metabolite production. At the species level, vertical inheritance and rare interspecies recombination events contributed to intraspecies BGC conservation (Fig. 1D). While HGT events likely introduced new BGCs into the genus, many of these were maintained over evolutionary timescales and thus contributed to species-level conservation (Fig. S4B at https://osf.io/entqc/). At the GCF level, frequent intraspecies recombination of small BGC sections (Table 1) can accelerate interspecies BGC diversification, allowing for species-specific gene gain and loss events (58) to alter patterns of expression and the products encoded by the pathway. We found that these evolutionary processes affected compound production, as exemplified by the *sta* and *sal* GCFs and the staurosporine and salinosporamide analogs produced, respectively (Fig. 3B). Finally, at the gene level, interspecies differences in nonsynonymous SNPs within the AT_1_ domain of the *salA* PKS gene were linked to the production of different analogs, revealing that minor genetic polymorphisms can also contribute to metabolite diversification. Evolutionary processes associated with vertical inheritance, operating at these three levels, promote interspecies differences in *Salinispora* specialized metabolism.

While genomic analyses offer insights into the evolutionary processes driving BGC evolution, the resulting metabolite is ultimately the biological trait undergoing selection. Surprisingly, we detected little evidence of selective sweeps operating within BGCs (Fig. S6A at https://osf.io/entqc/). One possible explanation is that genetic drift allows for neutral diversification, while selection maintains the production of advantageous compounds. For example, the *rif* GCF in S. arenicola accumulated a high degree of genetic polymorphisms yet maintained high fidelity for the production of the potent antibiotic rifamycin S. At the same time, we repeatedly detected the production of various analogs encoded by a single GCF, albeit at various intensities (Fig. 3B). These observations are consistent with recently proposed models of BGC evolution (59, 60) where nondirectional processes (e.g., genetic drift) allow for the exploration of ecological landscapes, while negative selection removes less favorable analogs. In this way, the exploitation of local environmental resources could drive population-specific pathway evolution as an initial step in species diversification. While correlations between microbial specialized metabolites and chemotaxonomy have been established (61, 62), linking these molecules to speciation events necessitates direct investigations into the evolutionary processes structuring the genetic diversity of populations.

### Conclusion.

There is growing appreciation that metabolite-mediated interactions can influence evolutionary fitness landscapes (15, 59). Here, we show that linkages between species delineations and BGC diversification had direct consequences on specialized metabolite production in the marine actinobacterial genus *Salinispora*. Our study highlights the role of vertical inheritance and the range of associated evolutionary processes that can alter BGC evolutionary trajectories. The long time frames in which many *Salinispora* BGCs are retained supports the concept that specialized metabolites play important functional roles in this taxon. Ultimately, resolving these roles will help inform our understanding of community dynamics in addition to uncovering how specialized metabolites contribute to the environmental distribution of microbes.

## MATERIALS AND METHODS

### Phylogenomic and flexible genome analyses.

We reanalyzed 118 *Salinispora* genomes (17) representing strains isolated from sponges and globally distributed marine sediments (96% of isolates). Previously, we assigned each strain to one of nine species based on genotypic and phenotypic characteristics (26) (Table S1 in the supplemental material). For each genome, protein-coding regions and gene annotations were assigned using Prokka v1.13.3 (63), and orthologs shared across all genomes were identified with Roary v3.12.0 (64) based on a minimum sequence identity of 85%. The resulting 2,106 potential orthologs were individually aligned using Clustal Omega (65) and screened for complete codon reading frames. The final 2,011 single-copy orthologs were concatenated to infer a core genome phylogeny using RAxML v8.2.10 (66) under the general time-reversal model with a gamma distribution for 100 replicates (Fig. 1A). Any orthologs not shared among all strains were assigned to the flexible genome. Species-specific flexible genes (defined as genes shared by all strains within a species but not observed in any other species) were assigned functional annotation with GhostKOALA against the nonredundant set of KEGG genes (67) for all *Salinispora* species with ≥3 genomes sequences.

### BGC identification and network visualization.

Each *Salinispora* genome was analyzed with antiSMASH v5.0-beta (68) to predict biosynthetic gene clusters (BGCs) associated with specialized metabolism. Identified BGCs were then analyzed with BiG-SCAPE v1.0.0 (69) (distance, 0.3) to determine relationships with experimentally characterized BGCs in the MIBiG database (70) and to group them into gene cluster families (GCFs). Pairwise distances (squared similarity scores ranging from 0 to 1) based on the BiG-SCAPE output were visualized as a network with a ForceAtlas layout in Gephi v0.9.2 (https://gephi.org/). The resulting network revealed that the GCFs generated by BiG-SCAPE split known BGCs into multiple GCFs (e.g., the lomaiviticin cluster in Fig. S3A at https://osf.io/entqc/ was split into two GCFs). Therefore, we used the BiG-SCAPE pairwise BGC distances to calculate the connectivity of the network (*M* = 0.927) in Gephi (set at a 1.0 resolution) and generated a second level of BGC clusters, or modules, to avoid separating BGCs that were predicted to produce related compounds.

To address the relative importance of geographic location and phylogenetic relatedness to *Salinispora* genetic diversity, we computed a Jaccard distance matrix using both flexible genome content and GCF composition. For the flexible genome, the distance matrix was visualized with a heatmap showing gene content similarity across strains (Fig. 1B). For GCF composition, the distance matrices for both GCFs and BGC modules were used to construct nonmetric multidimensional scales (NMDS) ordination plots. No major statistical differences were observed between the GCF and BGC module ordination analyses, and thus, only results from the GCF analysis are presented (Fig. 1D). Finally, the Jaccard dissimilarity matrices were used as input for a permutational multivariate analysis of variance (PERMANOVA) for 999 permutations.

### GCF evolutionary analyses.

For nine GCFs (Table S3), we extracted the BGC modules containing the respective MIBiG reference BGC from the network. Each BGC within the module was validated by screening for core biosynthetic genes known to be integral for product biosynthesis (Table S3). In addition, we performed a second verification for each GCF by applying a targeted search with BiG-SCAPE using the MIBiG reference BGC as the query (distance, 0.7). The BiG-SCAPE output was used for BGC visualizations and image generation (e.g., Fig. S9 at https://osf.io/entqc/).

We aligned all verified BGCs in a GCF with Mugsy (71) to generate a nucleotide alignment as the input for phylogenetic analysis using RAxML under the GTRGAMMA model with 100 replicates. The phylogenetic distances for each BGC were compared to core genome phylogenetic divergences calculated above for the same strain pairs (Fig. 2). The GCF phylogeny was used as input for the event inference parsimony model in Notung (72) by first rooting the phylogeny to minimize duplication and loss events for the top 10 parsimonious situations and reconciling each situation using a duplication (*D* = 2), transfer (*T* = 3), and loss (*L* = 1) score to achieve the most parsimonious outcome. Both the phylogeny and nucleotide alignment were then used to calculate the relative influence of recombination and mutation with ClonalFrameML (73). The *r/m* values (relative contribution of recombination to mutation) were calculated by incorporating the length and genetic distance of the recombining fragments and were compared to whole-genome alignments created from 28 representative genomes across the nine species. To construct whole-genome recombination networks (Fig. S5 at https://osf.io/entqc/), we identified recent gene flow events across all 118 *Salinispora* genomes using PopCOGenT (74), which uses a null model of sequence divergence to calculate a length bias to estimate recombining genomic segments.

To estimate synonymous to nonsynonymous substitution rates (*dN*/*dS*) among biosynthetic genes in the nine target GCFs, shared genes in each GCF were identified using CD-HIT-EST (75) at an 80% sequence identity threshold with the “most similar clustering” algorithm. Genes present in every genome encoding the BGC were aligned using Clustal Omega and used to calculate both *dN*/*dS* ratios and nucleotide diversity using the PopGenome package (76) in R. As a null comparison, we calculated the nucleotide diversity of the 2,011 core genes used to construct the core genome phylogeny above.

Salinosporamide AT domains were extracted from all strains containing the *sal* GCF (*n* = 30). Specifically, we extracted all protein domains within the BGC and filtered based on the “PKS_AT” annotation from the *salA* gene (GenBank accession no. EF397502). All AT domains were compared against the reference domain in S. tropica strain CNB440 (MIBiG accession no. BGC0000145) with BLAT (77) for further confirmation. Both AT_1_ (extender) and AT_L_ (loading) were initially included by aligning all AT domains with Clustal Omega and constructing a phylogeny with RAxML under the PROTGAMMABLOSUM62 protein model. After confirming AT_1_ and AT_L_ domains were phylogenetically distinct, we reran the phylogenetic analyses for the AT_1_ domains under identical parameters. AT domains from the *Streptomyces* BGCs that encode cinnabaramide (MIBIG accession no. BGC0000971) and avermectin were included for reference. The latter had the closest protein homology as identified by SWISS-MODEL (78) and was used as the template (PDB ID 4RL1) to generate a protein structure homology model of the AT_1_ domain in S. tropica for the most likely secondary structure under the DSSP program. All molecular graphic representations were visualized with PYMOL (https://pymol.org/2/).

### Strain cultivation and extraction.

Thirty representative *Salinispora* strains were grown in 50 ml of A1 media with the following formulation: 10 g/liter soluble starch, 4 g/liter yeast extract, 2 g/liter peptone, 10 g/liter CaCO_3_, and 22 g/liter instant ocean dissolved in deionized (DI) water. Cultures were supplemented with 5 ml/liter KBr (20 g/liter), 5 ml/liter Fe_2_(SO_4_)_3_ (8 g/liter), and shaken at 205 rpm at 28°C with metal springs to reduce clumping. After 1 week, strains were assigned as fast- or slow-growing based on visual cell densities. The slow-growing strains were transferred to new A1 media (minus CaCO_3_) and cultured under similar conditions for an additional week. All strains were then inoculated (0.5 ml) into triplicate 50-ml flasks containing A1 media supplemented as described above and shaken at 215 rpm at 30°C. After 4 days, 1 g of activated Amberlite XAD-7 resin was added to each flask (plus three media controls) to allow for passive adsorption of extracellular molecules. Cultures were extracted after 7 or 10 days (based on visual evidence of cell densities) with an equal volume of ethyl acetate. The organic layer was collected, dried with anhydrous Na_2_SO_4_, filtered, dried by rotary evaporation *in vacuo*, and stored at −20°C until further processing.

### Metabolomics and mass spectrometry.

Crude extracts were resuspended in HPLC-grade methanol (1 mg/ml) and filtered by centrifugation (0.2 μm). Samples were analyzed via liquid chromatography-tandem mass spectrometry (LC-MS/MS) with 1-μl injections into an Agilent 1290 HPLC system coupled to an Agilent accurate mass quadrupole time of flight (QTOF) spectrometer. For targeted analysis of the salinosporamides, 5-μl injections were also analyzed to ensure MS/MS fragmentation of the targeted compounds. Standards for lomaiviticin C; lymphostin; neolymphostin A; rifamycin W; salinipostins C, E, and H; salinosporamides A and B; and staurosporine were also analyzed. Parameters were set to a flow rate of 0.75 ml/min through a Kinetex C_18_ reversed-phase column (5 μm, 100 by 4.5 mm; Phenomenex, Torrence, CA, USA) with mobile-phase solvents A (water with 0.1% formic acid [FA] [vol/vol]) and B (acetonitrile with 0.1% FA [vol/vol]) and the following conditions: 0 to 4 min (95% solvent A, LC stream diverted to waste), 4 to 24 min (95% to 0% solvent A), 24 to 26 min (0% solvent A), 26 to 26.5 min (0% to 95% solvent A), and 26.5 to 30 min (95% solvent A). MS1 data were acquired in positive ion mode from 100 to 1,700 *m/z* with the top 5 most abundant precursor ions selected for MS2 fragmentation using a fixed collision energy of 30 eV from 50 to 1,700 *m/z*. Two MS2 scans were acquired per second, and ions were excluded from fragmentation for 30 s if acquired 3 times. MS data were collected with the following source parameters: nebulizer gas (nitrogen), 35 psig; drying gas flow of 11 liter/min; capillary voltage, 3,000 V; gas temperature, 300°C, acquiring 3 spectra/s. All data files were converted to mzXML using the MSConvert tool in the Trans-Proteomic pipeline (79) for further downstream analysis. Paired genomic and metabolomic data sets for each strain are available on the Paired Omics Data Platform (80).

Data files were preprocessed with MZmine v2.37.1 (81) with ion identity networking. Preprocessing parameters were set at a noise level of 1E3 for MS1 and 3E1 for MS2 scans. Chromatograms were compared using the ADAP chromatogram builder and deconvoluted using the local minimum search and isotopic peaks removed. Deconvoluted chromatograms were joined into an aggregated peak list with gap filling using an intensity tolerance of 10%, *m/z* tolerance of 0.0 *m/z* or 10 ppm, and a retention time tolerance of 0.2 min. For the ion identity network, the MetaCorrelate function was used with a minimum of five data points, two minimum data points on edge, a minimum feature shape correlation of 85%, and an *m/z* tolerance of 0.0 *m/z* or 10 ppm. For untargeted metabolomic analysis, mass spectrometry data files from the 1-μl injections were used to generate an MS1 feature table using MZmine adapted for ion identity networking. Data preprocessing parameters were generated from careful analysis of raw MS data. A final peak list was produced using the ADAP Chromatogram builder, quality filtering, chromatogram deconvolution, isotopic peak grouping, join alignment, gap filling, further quality filtering of gap-filled peaks, and ion identity networking.

MS1 molecular features were compared after normalization using a log_2_ transformation with Pareto scaling. A preliminary principal-components analysis ordination plot was generated for the 105 samples to assess data quality and ensure controls and standards were distinct from cultured strains. After manually removing all features found in standards and wash samples and subtracting feature intensities found in media controls, the remaining 3,168 features were normalized as above, and the intensities were averaged across the triplicate samples for each strain. A Euclidean distance matrix was generated and used to perform a permutational multivariate analysis of variance (82) with species as a fixed effect for 999 permutations under a reduced model. The average strain feature data were used to generate a final principal-components analysis ordination plot with 95% confidence intervals for each species represented by ellipses.

The MS2 classical molecular network was initially used to search for compounds encoded by the nine BGCs of interest by clusters containing either GNPS library matches or network nodes containing the standard compounds (Table S4). For sporolide, salinichelins, and salinilactams, where no standards were available, we manually curated the extracted ion chromatogram (EICs) at a maximum of 10 ppm error for the theoretical *m/z* of the molecules [M+H]^+^ and verified production if both the [M+H]^+^ and [M+Na]^+^ adducts were present in the MS1 chromatograms (Fig. S13 at https://osf.io/entqc/). Based on the results from the MS2 network and EICs, theoretical *m/z* [M+H]^+^ values for all analogs were correlated with the MS1 feature table based on *m/z* (Δppm, 2.2) and retention times (Table S4). After correcting for controls and low-quality hits, feature intensities were averaged across triplicate samples for each strain and validated using MS2 spectra from the classical molecular network (see above). Analog production was only confirmed if strains had intensity values corresponding to the MS1 feature table and were also identified in the MS2 classical molecular network. Using the analog intensities, a PERMANOVA was performed using a Euclidean distance matrix with species as a fixed effect for 999 permutations under a reduced model. A hierarchical clustering analysis was performed using the Euclidean distance matrix and visualized as a heatmap. All statistical analyses were performed in R.

For the salinosporamides, 5-μl LC-MS injections (at 1 mg/ml) were processed as above to generate a classical MS2 molecular network. The salinosporamide cluster was extracted using both the GNPS library matches and salinosporamides A and B standards (Fig. S10 at https://osf.io/entqc/). The relative production of salinosporamide analogs was calculated from the EICs at a maximum of 10 ppm error for the theoretical *m/z* of the molecules [M+H]^+^ and their adducts when above the 1E3 noise threshold level.

### Data availability.

All genomes are publicly available (Table S1). Public data sets for all metabolomic spectra files are available at https://massive.ucsd.edu (MSV000085890). All other data and relevant code used can be found at https://github.com/alex-b-chase/salBGCevol.
